# Cationic Liposomes with Different Lipid Ratios: Antibacterial Activity, Antibacterial Mechanism, and Cytotoxicity Evaluations

**DOI:** 10.3390/ph15121556

**Published:** 2022-12-14

**Authors:** Pengpeng Lu, Xinping Zhang, Feng Li, Ke-Fei Xu, Yan-Hong Li, Xiaoyang Liu, Jing Yang, Baofeng Zhu, Fu-Gen Wu

**Affiliations:** 1 Department of Emergency, The Second Affiliated Hospital of Nantong University, 6 North Hai’erxiang Road, Nantong 226001, China; 2 State Key Laboratory of Bioelectronics, School of Biological Science and Medical Engineering, Southeast University, 2 Sipailou Road, Nanjing 210096, China

**Keywords:** cationic liposome, antimicrobial, cytotoxicity, biocompatibility, bacteria

## Abstract

Due to their strong bacterial binding and bacterial toxicity, cationic liposomes have been utilized as effective antibacterial materials in many studies. However, few researchers have systematically compared their antibacterial activity with their mammalian cell cytotoxicity or have deeply explored their antibacterial and cytotoxicity mechanisms. Here, we prepared a series of cationic liposomes (termed CLs) using dimethyldioctadecylammonium chloride (DODAC) and lecithin at different molar ratios. CLs have the ability to effectively bind with Gram-positive and Gram-negative bacteria through electrostatic and hydrophobic interactions. Further, the CLs with high molar ratios of DODAC (30 and 40 mol%) can disrupt the bacterial wall/membrane, efficiently inducing the production of reactive oxygen species (ROS). More importantly, we carefully compared the antibacterial activity and the mammalian cell cytotoxicity of various CLs differing in DODAC contents and liposomal concentrations and revealed that, whether they are bacterial or mammalian cells, an increasing DODAC content in CLs can lead to an elevated cytotoxicity level. Further, there exists a critical DODAC contents (>20 mol%) in CLs to endow them with effective antibacterial ability. However, the variation in the DODAC content and liposomal concentration of CLs has different degrees of influence on the antibacterial activity or cytotoxicity. For example, CLs at high DODAC content (i.e., CL0.3 and CL0.4) could effectively kill both types of bacterial cells but only cause negligible toxicity to mammalian cells. We believe that a systematic comparison between the antibacterial activity and the cytotoxicity of CLs with different DODAC contents will provide an important reference for the potential clinical applications of cationic liposomes.

## 1. Introduction

With the abuse of conventional antibiotics in recent decades, the prevalence of multidrug-resistant bacteria has become a severe problem for human health [1]. To face this challenge, many novel nanomaterials have been fabricated by researchers, such as conventional metal- (e.g., Ag, Cu, Zn, and Ti) containing nanoagents [2,3,4,5], two-dimensional nanoagents [6,7], polymeric nanomaterials [8], micelles [9,10], nanovesicles [11,12], carbon dots [13,14], silicon nanoparticles [15,16], aggregation-induced emission (AIE) nanodots [17,18], nanocomposite materials [19,20], etc. The key advantage of antibacterial nanoparticles (NPs) is the high surface-to-volume ratio. Researchers can modify the surfaces of NPs with different functional moieties to endow these NPs with the capacity to inactivate bacteria via various mechanisms [21]. In addition, the nanomaterials with specific light- [22,23,24], heat- [25], electricity- [26], magnetic field- [27], and ultrasound- [28,29] responsive properties, as well as excellent antimicrobial activity, have also attracted growing interest from researchers.

Apart from the abovementioned antibacterial nanomaterials, liposome-based drugs also deserve proper attention [30]. Liposomes, which were first described in 1961 by Alec D. Bangham [31,32,33], are spherical vesicles with at least one lipid bilayer [34,35]. With a unique structure consisting of the lipid membrane and inner aqueous pool, liposomes can encapsulate both hydrophobic and hydrophilic drugs [36,37,38]. There is evidence that antibiotic-loaded liposomes exhibit synergistic efficacy of antibiotics and liposomes toward the bacteria [39,40,41,42]. For example, β-lactam antibiotics can suppress the biosynthesis of the bacterial cell wall [43] and quinolone antibiotics mainly inhibit topoisomerases IV and DNA gyrase [44]. Furthermore, liposomes have the ability to fuse with bacterial membranes [36] and directly release antibiotics inside the bacterial cells [45], which is the rationale behind the synergism between liposomes and antibiotics [46,47]. The fusion ability can be affected by several factors, such as bacterial membrane properties, divalent cations, bacterial surface pH, and temperature [48]. Other antibacterial materials, such as photosensitizers [49,50], fatty acids [51,52], and silver nanoparticles [53], can also be encapsulated in the inner aqueous pool or lipid bilayer to enhance the antibacterial efficiency. With the advantages of the targeting ability, long-term efficacy, improved drug stability, reduced drug toxicity, and extended circulation time, liposomes have been widely researched as an ideal drug delivery system [54,55]. Up until now, some liposome-based drugs have been approved by the United States Food and Drug Administration (FDA) and industrially produced in the antimicrobial field, such as Amikacin Liposome and Amphotericin B Liposome [56,57].

Cationic liposomes are a common class of liposomes that contains positively charged lipids. Besides the abovementioned features of liposomes, cationic liposomes have the additional capacity of targeting anionic sites [58] and a certain degree of cytotoxicity [59] due to their superficial positive charge [60]. Using the advantages of cationic liposomes, researchers have developed some valuable antibacterial materials [61,62,63]. However, few studies focus on the comparison between the antibacterial activity and mammalian cell toxicity of cationic liposomes.

**Scheme 1:**
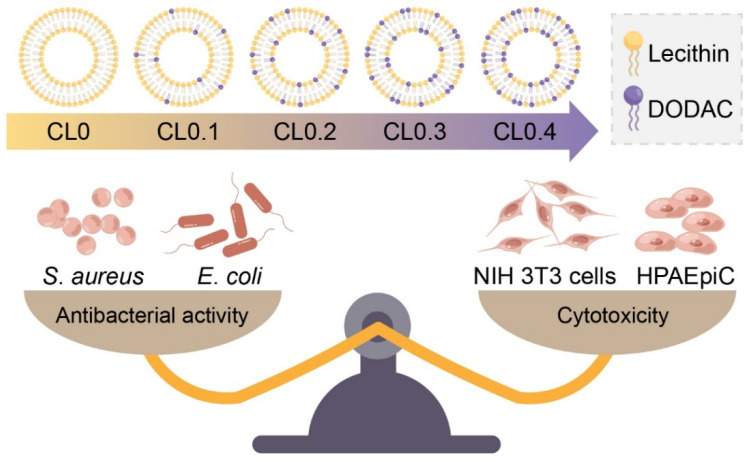
Scheme showing the structures of diverse CLs differing in various DODAC/lecithin molar ratios and the comparison between their antibacterial activity and cytotoxicity. CL0, CL0.1, CL0.2, CL0.3, and CL0.4 in the scheme represent the CLs with a DODAC content of 0, 10, 20, 30, and 40 mol%, respectively.

Here, we fabricated a series of cationic liposomes (termed CLs) through ultrasonication using different molar ratios of lecithin and dimethyldioctadecylammonium chloride (DODAC) (Figure S1). The contents of DODAC were 0, 10, 20, 30, and 40 mol% (termed CL0, CL0.1, CL0.2, CL0.3, and CL0.4, respectively). Lecithin, as the phosphatidylcholine (PC) fraction, is common and easily available. DODAC, containing a quaternary ammonium group and two long hydrocarbon chains (each has 18 carbon atoms) is a commonly used cationic surfactant whose structure is similar to that of a phospholipid. Herein, we reported the preparation, characterization, and antimicrobial mechanism of these cationic liposomes. Most importantly, we also carefully compared the antibacterial effect and the cytotoxicity toward mammalian cells of different CLs with various DODAC contents and liposomal concentrations (Scheme 1).

## 2. Results and Discussion

### 2.1. Characterization of CLs

**Figure 1:**
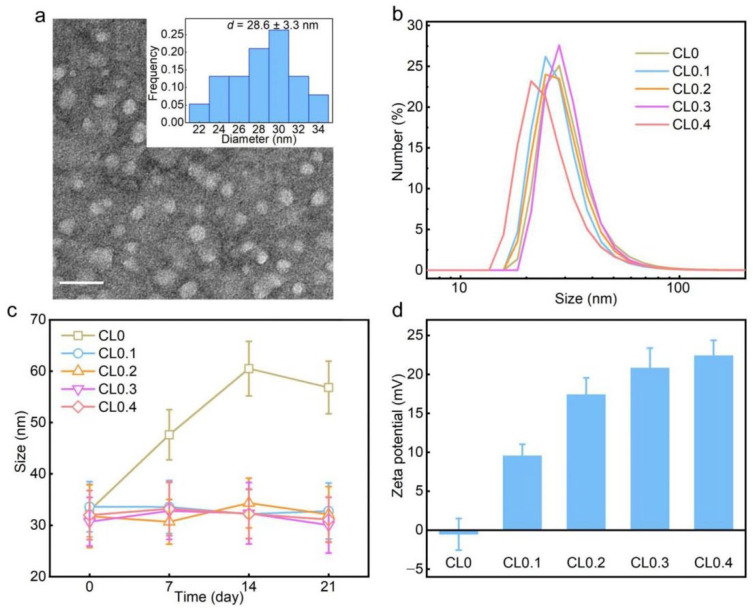
Characterization of CLs. (**a**) TEM image of CL0.4 and corresponding size distribution histogram (inset). Scale bar = 50 nm. (**b**) Hydrodynamic diameter distributions of CL0, CL0.1, CL0.2, CL0.3, and CL0.4 measured by DLS. (**c**) Changes of the hydrodynamic diameters of different CLs within 3 weeks. (**d**) Zeta potentials of CLs in PBS.

As shown in the transmission electron microscopy (TEM) results (Figure 1a and Figure S2), the obtained CLs (CL0, CL0.1, CL0.2, CL0.3, and CL0.4) had similar sizes (26–32 nm) and spherical structures. The hydrodynamic diameters measured by dynamic light scattering (DLS) were about 27–32 nm (Figure 1b), which agreed well with the average sizes revealed by TEM. Moreover, the polydispersity indexes (PDI) of CLs were all below 0.3, which showed their relatively eligible aqueous dispersity. As shown in Figure 1c, the hydrodynamic diameter of CL0 had an obvious rise upon prolonged storage (7, 14, and 21 d); in contrast, the hydrodynamic diameters of other groups (CL0.1, CL0.2, CL0.3, and CL0.4) only had slight fluctuations. The results revealed that the CLs containing DODAC had improved colloidal stability. To investigate the surface charges of CLs, we measured the zeta potentials of CLs in a phosphate-buffered saline solution (PBS; pH 7.4) (Figure 1d). Except for the group of CL0 that had a close-to-neutral zeta potential value, all the liposomes were positively charged (10–22 mV), which may explain the excellent colloidal stability of the CLs and ensure their potential interaction with negatively charged bacteria via electrostatic attraction. Additionally, differential scanning calorimetry (DSC) was conducted for the phase-state identification of CLs. The data (Figure S3) showed that CL0.4 was found in the liquid-crystalline (fluid) phase from 0 °C to 50 °C. Since lecithin has a phase transition temperature below −10 °C, all the CL samples (CL0–CL0.4) were in the fluid phase.

### 2.2. Antibacterial Activity of CLs

**Figure 2:**
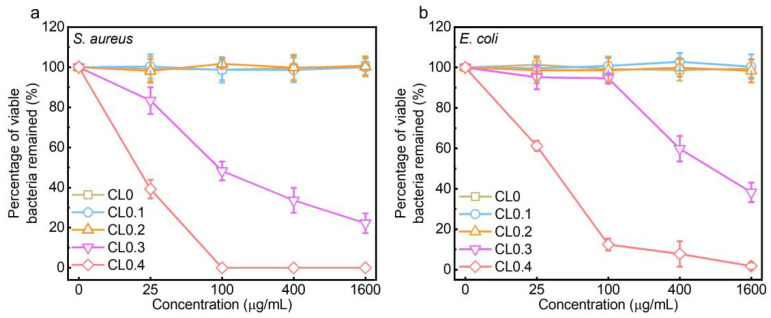
Antibacterial activities of CLs. Line charts showing viable bacterial percentages of *S*. *aureus* (**a**) and *E*. *coli* (**b**) after being treated with CL0, CL0.1, CL0.2, CL0.3, or CL0.4 at various concentrations for 5 h.

We selected *Staphylococcus aureus* (*S*. *aureus*) and *Escherichia coli* (*E*. *coli*) as the representatives of Gram-positive and Gram-negative bacteria, respectively. The agar plate assay, a classical bacterial counting method, was applied to evaluate the antibacterial effect of CLs, and the corresponding photographs and statistical results were shown in Figure 2, Figures S4 and S5. It could be seen that the content of DODAC and the total liposomal concentration both played an important role in the antibacterial performance of CLs. Specifically, at a low concentration of DODAC (i.e., 0, 10, and 20 mol%), the CLs exhibited negligible antibacterial effect toward both types of bacteria, even at a total liposomal concentration of up to 1600 μg/mL. When the DODAC content increased to 30 mol%, the CLs (CL0.3) could elicit a dose-dependent antibacterial effect toward the two types of bacteria. However, for CL0.3, even at the highest concentration of 1600 μg/mL, it could not completely kill all the bacterial cells. Furthermore, increasing the DODAC content to 40 mol% (i.e., the CL0.4 sample) achieved a complete killing effect toward the *S*. *aureus* bacteria at a concentration of 100 μg/mL or higher. We also noted that, for *S*. *aureus*, the DODAC weight content of CL0.2 at 1600 μg/mL was 7.6 times that of CL0.4 at 100 μg/mL. However, in sharp contrast to CL0.2 at 1600 μg/mL which did not show any antibacterial effect, CL0.4 at 100 μg/mL killed almost all the bacteria. Collectively, there might exist a threshold value (>20 mol%) of the DODAC content of CLs to realize a notable antibacterial effect. In addition, by comparing Figure 2b with Figure 2a, we reveal that the *E*. *coli* bacteria showed stronger resistance to CLs than the *S*. *aureus* bacteria. Even at the highest concentration of 1600 μg/mL, CL0.4 could not completely kill all the *E*. *coli* bacteria (Figure 2b and Figure S5). The different drug-tolerance levels of the two types of bacteria may be due to their different cell wall/membrane structures. We also evaluated the antibacterial activity of free DODAC at the concentrations covering the DODAC concentrations of all CL samples for bacterial and mammalian cell treatment (Figure S6). It was found that the antibacterial activity of DODAC was inhibited when it was applied in the liposomal format, which may be attributed to the incomplete release of DODAC from CLs.

### 2.3. Antibacterial Mechanisms of CLs

**Figure 3:**
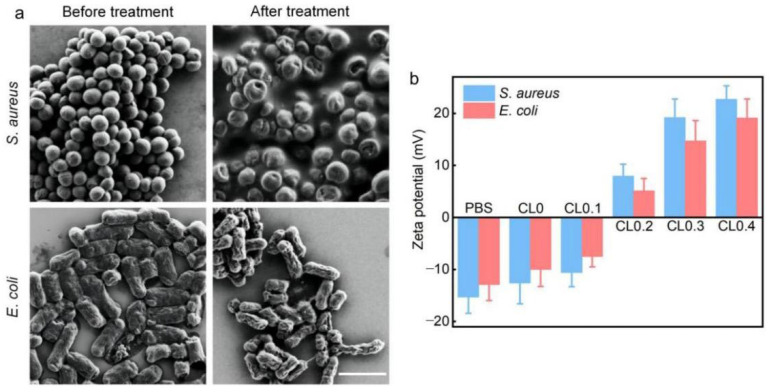
(**a**) SEM images of *S*. *aureus* and *E*. *coli* cells before and after the treatment of CL0.4 for 5 h. Scale bar = 2 μm. (**b**) Zeta potentials of *S*. *aureus* and *E*. *coli* bacteria after treatment with PBS or different CLs for 5 min in PBS.

To reveal the antibacterial mechanisms of the CLs toward the two types of bacteria, we adopted scanning electron microscopy (SEM) to observe the bacterial morphology before and after the treatment with CL0.4. As presented in Figure 3a, the bacterial cells after CL0.4 treatment were slightly or severely damaged with surface wrinkles or holes. To further explore the antibacterial mechanism of CLs, we conducted the following experiments. First, the zeta potential changes of *S*. *aureus* and *E*. *coli* bacteria after treatment with CLs in PBS were measured and shown in Figure 3b. The zeta potentials of *S*. *aureus* and *E*. *coli* without CL treatment were −15.3 and −12.9 mV, respectively. With the increase of DODAC content (from 0 to 10 mol%), the zeta potentials of the bacteria gradually became less negatively charged. In the groups with high DODAC content (20, 30, and 40 mol%), the potentials even turned positive. Specifically, for the CL0.4 group, the zeta potentials of the *S*. *aureus* and *E*. *coli* were 22.7 and 19.1 mV, respectively. The above results indicated that the positively charged CLs could be adsorbed onto the bacteria via electrostatic interaction, thus changing the surface charges of the bacterial cell. In addition, after the electrostatic adsorption of the cationic CLs onto the negatively charged bacterial surface, the CLs may spread on the bacterial surface or fuse with the bacterial cell wall/membrane. Therefore, the hydrophobic hydrocarbon chains of DODAC may be exposed for further hydrophobic interaction with the bacterial cells.

**Figure 4:**
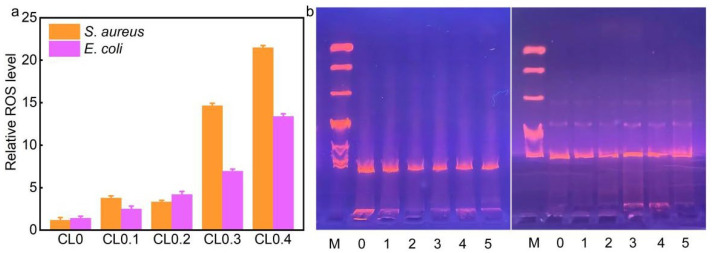
(**a**) Relative ROS levels in *S*. *aureus* and *E*. *coli* bacteria after incubation with various CLs for 5 h. (**b**) Agarose gel electrophoresis results of the extracted DNA from *S*. *aureus* (left panel) or *E*. *coli* (right panel) cells treated with PBS (control) or various CLs. M: DNA Ladder DL10000. 0, 1, 2, 3, 4, and 5 represent *S*. *aureus* and *E*. *coli* treated with PBS (control), CL0, CL0.1, CL0.2, CL0.3, CL0.4, and CL0.5, respectively.

Next, we adopted the reactive oxygen species (ROS) probe, 2′,7′-dichlorodihydrofluorescein diacetate (DCFH-DA), to detect the production of cellular ROS in *S*. *aureus* and *E*. *coli* bacteria. DCFH-DA has no fluorescence, but when it is hydrolyzed by intracellular esterases, it can be converted into 2′,7′-dichlorodihydrofluorescein (DCFH), which can further be oxidized by ROS into fluorescent 2′,7′-dichlorofluorescein (DCF). As shown in Figure 4a, the ROS levels of the CL0.3- and CL0.4-treated *S*. *aureus* cells were 14.6- and 21.5-fold higher, respectively, than that of the control group. For *E*. *coli* bacteria, the ROS levels of the CL0.3- and CL0.4-treated groups were 6.9- and 13.4-fold higher than that of the control group, respectively. In the other groups (CL0, CL0.1, and CL0.2), the production of ROS only had a slight rise. The levels of ROS generation closely agreed with the antibacterial effects of various CLs, indicating that ROS generation is an important antibacterial mechanism of CLs toward the two types of bacteria. Finally, we extracted the DNA molecules from *S*. *aureus* and *E*. *coli* cells that were treated with PBS (control) or various CL samples. Then, these DNA molecules were analyzed by the agarose gel electrophoresis assay. As shown in Figure 4b, the extracted DNA from the untreated and CLs-treated bacterial groups showed similar bands, suggesting that CLs did not have a noticeable damaging effect on the DNA molecules of the bacteria.

Collectively, based on the above analyses, it could be seen that the destruction of bacterial walls/membranes and the production of ROS were the two main factors that can account for the inactivation of the two types of bacteria.

### 2.4. Cytotoxicity of CLs

**Figure 5:**
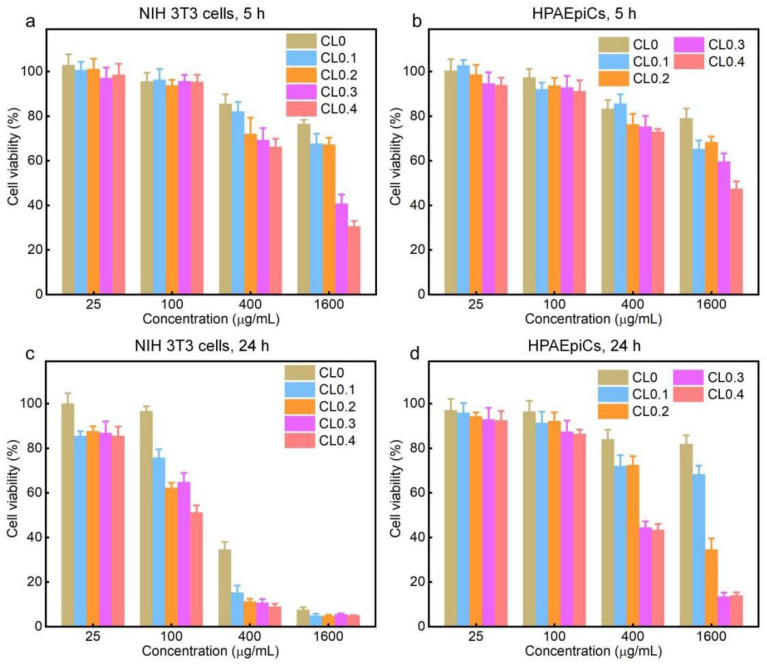
Relative viabilities of NIH 3T3 cells and HPAEpiCs after incubation with different CLs for 5 and 24 h. (**a**) NIH 3T3 cells, 5 h, (**b**) HPAEpiCs, 5 h, (**c**) NIH 3T3 cells, 24 h, (**d**) HPAEpiCs, 24 h.

We then tested the cytotoxicity of various CLs toward two normal mammalian cells—NIH 3T3 cells (mouse embryo fibroblast cells) and HPAEpiCs (human pulmonary alveolar epithelial cells). We selected two incubation time points: 5 h (which is the time period for antibacterial treatment) and 24 h (which is the most commonly used time period for cytotoxicity evaluation). As shown in Figure 5a–d, the 3-(4,5-dimethyl-2-thiazolyl)-2,5-diphenyl-2*H*-tetrazolium bromide (MTT) assay results demonstrated that the viabilities of the two types of cells decreased with the increase of DODAC content, liposomal concentration, and incubation time. In addition, the cytotoxicity of CLs to HPAEpiCs was lower than that to NIH 3T3 cells, which may be due to the better resistance to environmental changes of epithelial cells. Compared with the above antibacterial results shown in Figure 2, CL0.3 and CL0.4, which can kill most of or even all the bacterial cells at 100 μg/mL for 5 h, did not show evident mammalian cell toxicity after incubation for the same time period of 5 h, indicating that CLs can selectively kill bacterial cells while leaving the mammalian cells only slightly affected or unaffected, which suggested their potential for practical in vivo applications. Notwithstanding, when the concentration of CLs reached 400 μg/mL or higher, the CLs exhibited a certain degree of cytotoxicity to mammalian cells after incubation for 5 h, which illustrates that the in vivo working concentration of CLs must be carefully optimized. Besides, when the incubation time was increased to 24 h, the mammalian cell toxicity had a notable increase, especially after treatment with high-concentration liposomes (400 or 1600 μg/mL). Further, we also conducted the cytotoxicity test of free DODAC to NIH 3T3 cells (Figure S6). At the same content of DODAC, CLs had a lower cytotoxicity than free DODAC.

**Figure 6:**
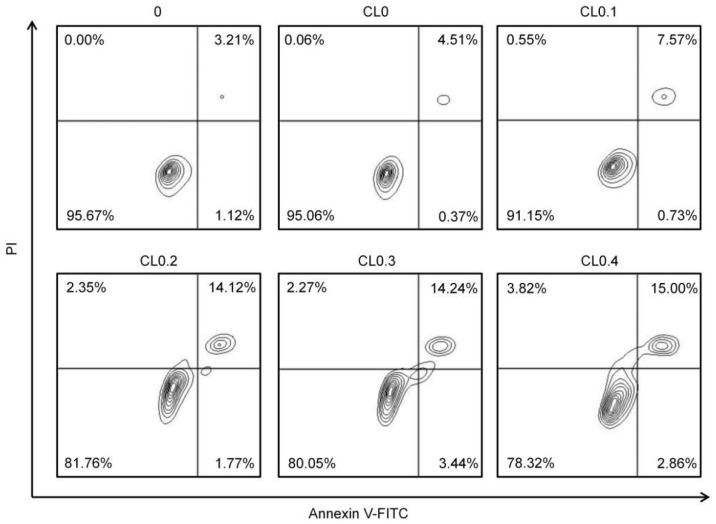
Flow cytometry analysis results of NIH 3T3 cells after treatment with the culture medium (control) or various concentrations of CLs (400 μg/mL) for 5 h. Before analysis, the cells were stained with annexin V-FITC and PI.

Next, we used the annexin V-fluorescein isocyanate (FITC)/propidium iodide (PI) apoptosis detection kit to analyze the role of apoptosis in the CL-induced cytotoxicity. Before being stained, the NIH 3T3 cells were treated with various CLs at 400 μg/mL. As shown in Figure 6, with the increasing DODAC contents, the proportion of apoptotic cells increased, illustrating that DODAC plays an important role in inducing cell apoptosis.

## 3. Materials and Methods

### 3.1. Materials

Lecithin, DODAC, and MTT were purchased from Shanghai Aladdin Biochemical Technology Co., Ltd. (Shanghai, China). Dimethyl sulfoxide (DMSO) and chloroform were bought from Sinopharm Chemical Reagent Co., Ltd. (Shanghai, China). ROS Assay Kit was purchased from KeyGEN BioTECH Co., Ltd. (Nanjing, China). The TIANamp Bacteria DNA Kit was bought from Tiangen Biotech (Beijing) Co., Ltd. (China). All other chemicals were of analytical reagent grade and used without further purification. All solutions/suspensions were prepared with deionized water (18.2 MΩ cm) purified by a Milli-Q water-purification system (Billerica, MA, USA).

### 3.2. Preparation of CLs

Briefly, the same total mass of lecithin and DODAC were mixed at different molar ratios in the chloroform. The molar contents of DODAC were 0, 10, 20, 30, and 40 mol% (termed CL0, CL0.1, CL0.2, CL0.3, and CL0.4, respectively). Next, the obtained mixtures were blown dry by nitrogen gas and were then put in the vacuum drying oven overnight to completely remove the chloroform. After that, the obtained lipid dry films were added with PBS, which were subjected to heating treatment at 65 °C (accompanied with intermittent vortexing) for 20 min and subsequent probe sonication at 25% power for 2 min (consisting of 20 cycles, with a 2 s pause after 4 s working per cycle). The obtained suspensions were stored at 4 °C for further use. These liposomal suspensions with a fixed lipid concentration (4 mg/mL) and varied DODAC contents were diluted to achieve various samples with different lipid concentrations (25, 50, 100, 200, 400, 800, 1600, or 3200 μg/mL) for further experiments.

### 3.3. Characterization

For TEM measurements, the suspensions of CLs (200 μg/mL) were deposited onto the carbon-coated grid and then negatively stained by 2% phosphotungstic acid solution. After the CLs were dried at room temperature, their size and morphology were characterized by TEM (JEM-2100, JEOL Ltd., Akishima, Japan). To determine the hydrodynamic diameters and zeta potentials of CL samples, the samples at a concentration of 400 μg/mL were measured by a Nano ZS zetasizer instrument (Malvern Instruments, Worcestershire, UK). To prepare the samples for DSC measurements, DODAC (~8 mg) was added with 32 μL deionized water, and the resulting mixture was subjected to repeated (at least four times) heating and cooling between −20 and 60 °C for obtaining the fully hydrated sample. For the preparation of CL0.4, ~8 mg lipid powder was dissolved in chloroform, blown dry with nitrogen gas, and further dried under vacuum to obtain the dry lipid film. Next, the obtained dry lipid film was added with 32 μL deionized water and then subjected to repeated (at least four times) heating and cooling between −20 and 60 °C. Finally, the DSC curves of the two samples were collected using a DSC instrument (DSC 214 Polyma, Netzsch, Selb, Germany) with a heating rate of 1 °C/min from −10 °C to 60 °C.

### 3.4. Culture of Bacterial Cells

*S*. *aureus* and *E*. *coli* bacterial cells (purchased from the China Center of Industrial Culture Collection (CICC), Beijing, China) were cultured in lysogeny broth (LB) media in a shaking incubator (160 rpm) at 37 °C.

### 3.5. Agar Plate Count Assay

To evaluate the antibacterial activity of CLs against *S*. *aureus* and *E*. *coli*, we mixed the bacterial suspensions (optical density at 600 nm (OD_600_): ~0.1; 200 μL) with 0, 50, 200, 800, or 3200 μg/mL of various CL suspensions (including CL0, CL0.1, CL0.2, CL0.3, and CL0.4) (200 μL), and incubated the resultant mixtures for 5 h in a shaking incubator (160 rpm) at 37 °C. After the treatments, the bacterial suspensions were diluted (with a dilution ratio of 1:4000 or 1:8000), plated (50 μL) on LB agar plates with the help of small glass balls, and further incubated at 37 °C for 16 h. Finally, the number of the bacterial colonies on the plates were counted.

### 3.6. SEM Observation of Bacteria

The bacterial suspensions (OD_600_: ~0.5; 1 mL) were centrifuged at 8000 rpm for 5 min and then treated with PBS or 4 mg/mL CL0.4 suspension (1 mL) for 5 h in a shaking incubator (160 rpm) at 37 °C. Then, all the samples were centrifuged at 8000 rpm for 5 min to remove the supernatants and fixed with 2.5 vol% glutaraldehyde solution (solvent: PBS) at 4 °C overnight. After that, the bacteria were washed with PBS, and then sequentially treated with 30%, 50%, 70%, 80%, 90%, and 100% ethanol solutions. In each step of dehydration, the sample was suspended in the respective ethanol solution, incubated for 15 min, and centrifuged at 8000 rpm for 5 min. Finally, the bacterial suspensions in 100% ethanol solutions were deposited onto polished silicon wafers separately and allowed to dry for SEM observation.

### 3.7. Zeta Potential Measurements of Bacterial Suspensions

*S*. *aureus* or *E*. *coli* bacterial suspensions (OD_600_: ~0.1; 500 μL) were mixed with PBS (pH 7.4; 500 μL) or 800 μg/mL of various CLs (dispersed in PBS, pH 7.4; 500 μL), and the resultant mixtures were incubated in a shaking incubator (160 rpm) at 37 °C for 5 min. Next, the mixtures were centrifuged and resuspended in PBS for two times. Finally, the zeta potentials of the bacteria without and with CL treatment were measured using the zetasizer instrument.

### 3.8. Analysis of Intracellular ROS Generation

The bacterial suspensions (OD_600_: ~0.1; 500 μL) were mixed with PBS (500 μL) or various CLs at a liposomal concentration of 800 μg/mL (500 μL) for 5 h in a shaking incubator (160 rpm) at 37 °C. After that, 1 μL of DCFH-DA (Beyotime Biotechnology, Inc., Shanghai, China) solution (10 mM) was added to each sample (1 mL), and the obtained mixtures were further incubated in the dark at room temperature for 20 min. Then, the bacteria were washed three times with PBS, and the ROS were measured using a flow cytometer (NovoCyte 2070R, ACEA Biosciences Inc., San Diego, CA, USA) at an excitation wavelength of 488 nm.

### 3.9. DNA Extraction and Agarose Gel Electrophoresis Assay

The bacterial cells (OD_600_: ~0.5; 1 mL) were centrifuged at 10,000 rpm for 1 min and then treated with PBS (1 mL) or various CLs (4 mg/mL; 1 mL) for 5 h in a shaking incubator (160 rpm) at 37 °C. Next, according to the instructions of the FastPure Bacteria DNA Isolation Mini Kit, the DNA molecules were extracted from *S*. *aureus* and *E*. *coli* samples. Then, these DNA samples were added with 10× loading buffer solutions. After this step, the DNA Ladder, DL10000 (purchased from Takara Biomedical Technology Co., Ltd., Beijing, China), was set as the DNA marker, and the DNA samples of bacteria were separated on a 0.7 wt% agarose gel plate. The agarose gel electrophoresis was allowed to run for ~60 min at a fixed voltage of 100 V at room temperature using an electrophoresis apparatus (Tanon EPS 300, Tanon Science & Technology Co., Ltd., Shanghai, China). Finally, a gel imaging system (Tanon 3500R, Tanon Science & Technology Co., Ltd., Shanghai, China) was used to image the above agarose gel plate.

### 3.10. MTT Assay

To evaluate the cytotoxicity of CLs, NIH 3T3 cells were cultured in Dulbecco’s modified Eagle’s medium (DMEM) in a humid incubator at 37 °C and 5% CO_2_. The cells were seeded in a 96-well plate at a density of 5 × 10^3^ cells per well and incubated overnight. The cells were then incubated with various concentrations (0, 25, 100, 400, and 1600 μg/mL) of CLs for 5 h or 24 h. After that, each well was added with 10 μL of MTT solution (5 mg/mL) and incubated for 4 h, followed by the addition of 150 μL DMSO. Finally, cell viability was determined via measuring the absorbance at 570 nm using the Multiskan FC microplate photometer (Thermo Scientific, Multiskan FC, Waltham, MA, USA). HPAEpiCs were also chosen for cytotoxicity evaluation and were cultured in the Roswell Park Memorial Institute (RPMI) 1640 medium. The other steps were the same as those for NIH 3T3 cells. NIH 3T3 cells and HPAEpiCs were obtained from GuangZhou Jennio Biotech Co., Ltd. (Guangzhou, China).

### 3.11. Apoptosis Assay

NIH 3T3 cells were plated in a 6-well plate at a density of 5 × 10^4^ cells/mL. After being cultured for 24 h, the cells were treated with culture medium (control) or various CLs at a liposomal concentration of 400 μg/mL for 5 h and then digested with trypsin without ethylene diamine tetraacetic acid (EDTA) disodium salt. After being washed with PBS for two times, these cells were treated with the FITC-annexin V/PI apoptosis kit (UElandy, Suzhou, China). Finally, these samples were analyzed by flow cytometry.

## 4. Conclusions

In summary, we successfully prepared a series of CLs by mixing different molar ratios of DODAC and lecithin. Benefiting from the two hydrocarbon chain-containing quaternary ammonium group of DODAC, the CLs with high DODAC contents (CL0.3 and CL0.4) could effectively bind to both Gram-positive and Gram-negative bacteria via electrostatic and hydrophobic interactions and then inactivate both of them. The antibacterial mechanism of CLs against the two types of bacteria included the disruption of bacterial cell wall/membrane and the generation of ROS. Furthermore, we systematically compared the antibacterial activity and mammalian cell cytotoxicity of the CLs at different liposomal concentrations. First, both the antibacterial activity and mammalian cell cytotoxicity of CLs increased with an increasing DODAC content and liposomal concentration. Second, it was found that there was a threshold value of DODAC content of 20 mol%, above which the CLs could effectively or even completely kill all the bacteria. Third, CLs were found to be able to decrease the biotoxicity of DODAC, whether for bacteria or mammalian cells. Finally, it was revealed that the DODAC contents and liposomal concentrations had varying degrees of influence on the antibacterial activity and the cytotoxicity of CLs, and the CLs at high DODAC contents (i.e., CL0.3 and CL0.4) could selectively kill both types of bacterial cells while only causing negligible toxicity to the mammalian cells, indicating their suitability for potential biomedical applications. These results demonstrated that when using CLs as the antibacterial drug-carrying platform, their inherent features should be considered. In this study, we chose a commonly used cationic lipid DODAC to prepare the CLs. Besides DODAC, other similar-structured lipids with different hydrocarbon chain lengths, such as dimethyldihexadecylammonium chloride (DHDAC) and dimethylditetradecylammonium chloride (DTDAC), can also be used to fabricate CLs. Considering that the CLs can interact with bacteria and mammalian cells through electrostatic interaction and/or hydrophobic interaction, the cationic lipid components (DODAC, DHDAC, and DTDAC) of CLs, which possess the same charge (due to their single quaternary ammonium group) and different hydrocarbon chain lengths, may have a similar effect on the electrostatic interaction between CLs and cells. However, the different hydrophobic hydrocarbon chain lengths of these cationic lipids may affect the hydrophobic interaction of CLs with bacteria or mammalian cells. Specifically, the cationic lipids with shorter hydrocarbon chains may have a stronger tendency to fuse with cells and thereby promote the antibacterial activity and the mammalian cell toxicity of CLs. In brief, our work provides an essential support for future research in this field and may contribute to further development of CLs for diverse clinical applications.

## Data Availability

All the data are contained within the article and Supplementary Material.

## Supplementary Materials

The following supporting information can be downloaded at: https://www.mdpi.com/article/10.3390/ph15121556/s1, other experimental details (including Figure S1: Chemical structures of lecithin and DODAC. Figure S2: TEM images (scale bars: 50 nm) of CL0, CL0.1, CL0.2, and CL0.3, and corresponding size distribution histograms (insets). Figure S3: DSC curves of free DODAC and CL0.4 suspensions. Figures S4 and S5: Agar plate photographs of *S*. *aureus* or *E*. *coli* bacterial colonies formed from the corresponding cells treated with CL0, CL0.1, CL0.2, CL0.3, or CL0.4 at 0, 25, 100, 400, or 1600 μg/mL for 5 h. Figure S6: Relative viabilities of *S*. *aureus*, *E*. *coli*, and NIH 3T3 cells after incubation with different concentrations of DODAC for 5 h).
