# Supplementary material for: Cationic Liposomes with Different Lipid Ratios: Antibacterial Activity, Antibacterial Mechanism, and Cytotoxicity Evaluations
Source: Pharmaceuticals (Basel). 2022 Dec 14;15(12):1556. doi: 10.3390/ph15121556 (PMC9783835; doi:10.3390/ph15121556)
Supplement: Supplementary file 1 [file pharmaceuticals-15-01556-s001.zip › pharmaceuticals-2012578-supplementary.pdf]

**Supporting Information**

# Cationic Liposomes with Different Lipid Ratios: Antibacterial Activity, Antibacterial Mechanism, and Cytotoxicity

*Pengpeng Lu,[1,†] Xinping Zhang,[2,†] Feng Li,[1] Ke-Fei Xu,[2] Yan-Hong Li,[2] Xiaoyang Liu,[2] Jing Yang,[2] Baofeng Zhu,[1,]* and Fu-Gen Wu[1,2,]**

*[1]Department of Emergency, The Second Affiliated Hospital of Nantong University, 6 North Hai'erxiang Road, Nantong, Jiangsu 226001, P. R. China*

*[2]State Key Laboratory of Bioelectronics, School of Biological Science and Medical Engineering, Southeast University, 2 Sipailou Road, Nanjing, Jiangsu 210096, P. R. China*

**E-mail: bfzhunt@163.com (B.Z.)*

**E-mail: wufg@seu.edu.cn (F.G.W.)*

*[†] These authors contributed equally to this work.*

**Supplementary figures**

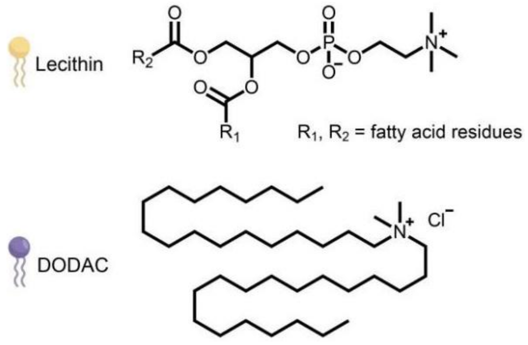


**Figure S1.** Chemical structures of lecithin and DODAC.

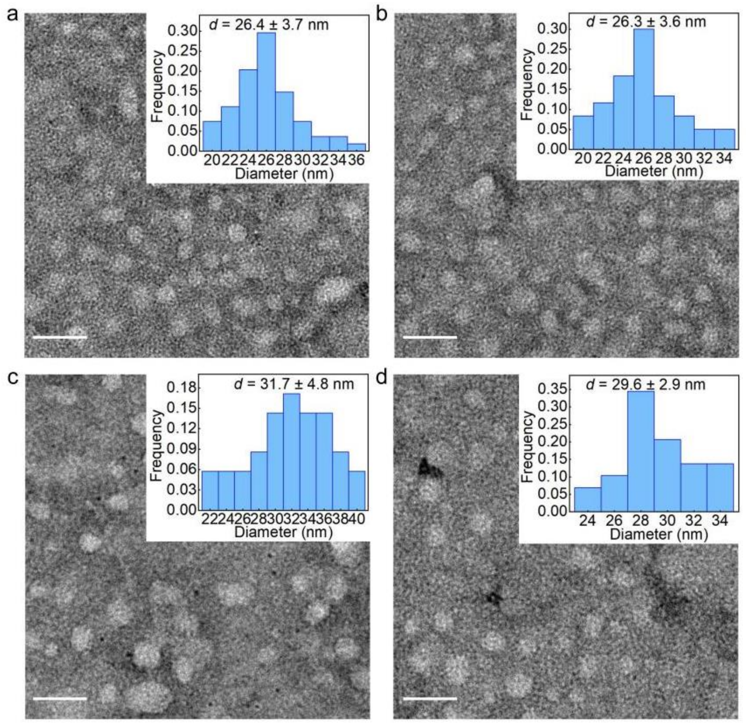


**Figure S2.** Transmission electron microscopy (TEM) images (scale bars: 50 nm) of CL0 (a), CL0.1 (b), CL0.2 (c), and CL0.3 (d), and corresponding size distribution histograms (insets).

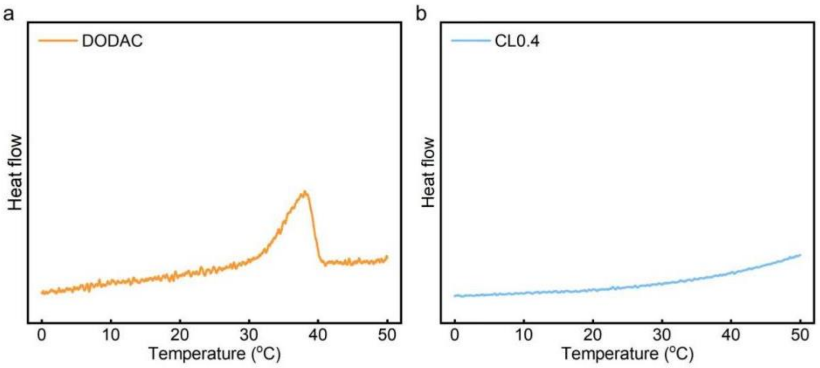


**Figure S3.** DSC curves of free DODAC (a) and CL0.4 (b) suspensions.

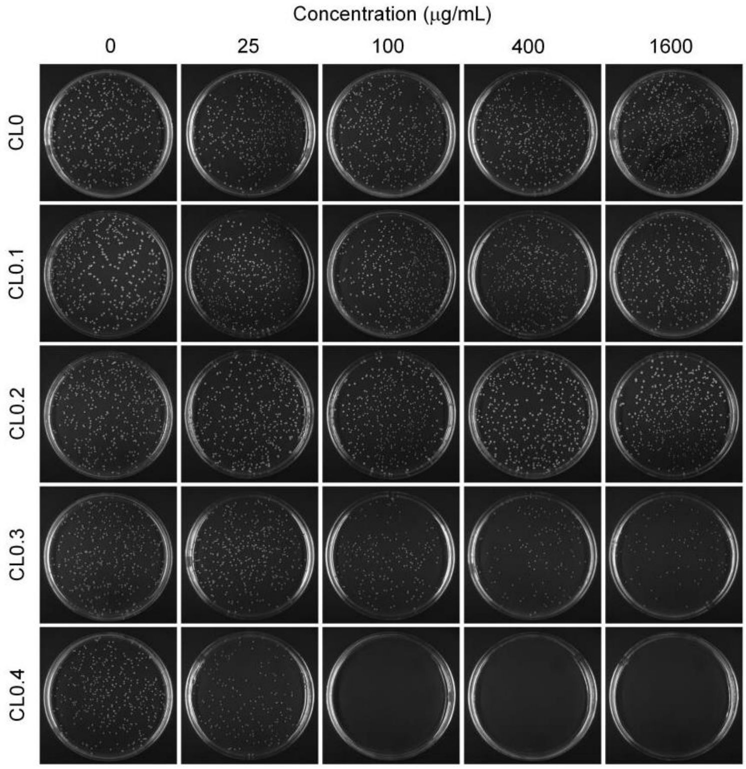


**Figure S4.** Agar plate photographs of *S. aureus* bacterial colonies. Before being plated on agar media, the bacterial samples were treated with CL0, CL0.1, CL0.2, CL0.3, or CL0.4 at 0, 25, 100, 400, or 1600 μg/mL for 5 h. The dilution time of the bacterial suspensions was 4000.

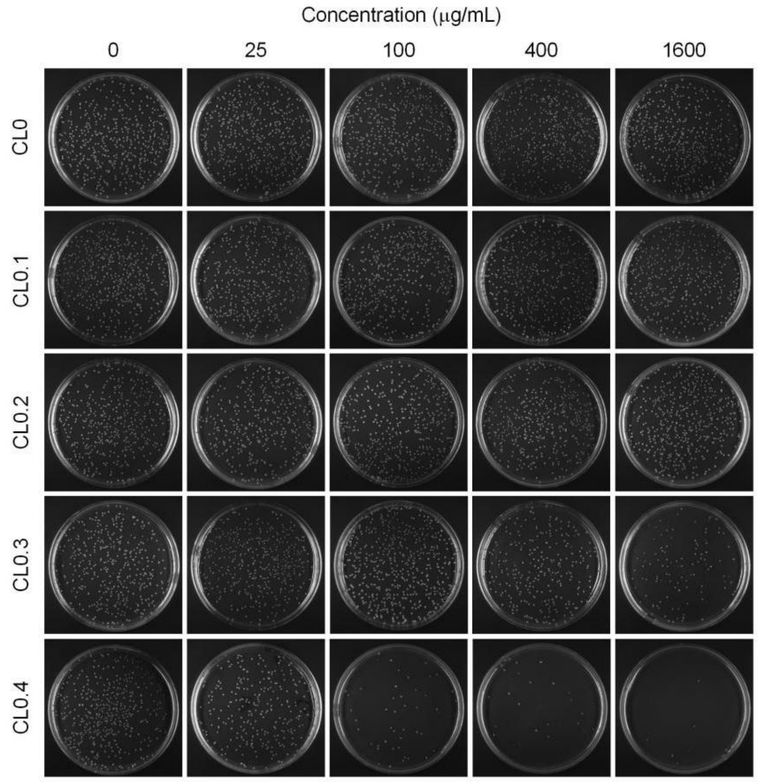


**Figure S5.** Agar plate photographs of *E. coli* bacterial colonies. Before being plated on solid media, the bacterial samples were treated with CL0, CL0.1, CL0.2, CL0.3, or CL0.4 at 0, 25, 100, 400, or 1600 μg/mL for 5 h. The dilution time of the bacterial suspensions was 8000.

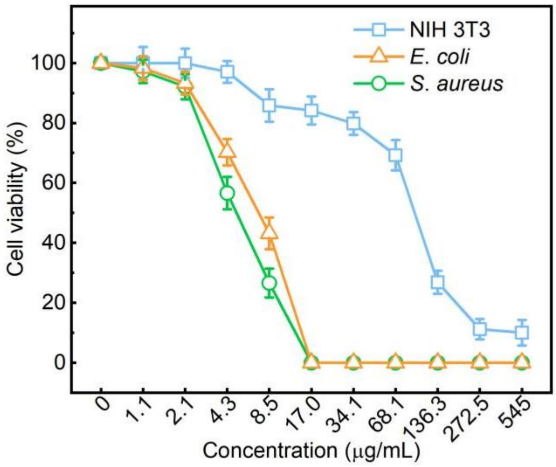


**Figure S6.** Relative viabilities of *S. aureus*, *E. coli*, and NIH 3T3 cells after incubation with different concentrations of DODAC for 5 h.
